# Rapid automatized naming skills of children with intellectual disability

**DOI:** 10.1016/j.heliyon.2021.e06944

**Published:** 2021-05-03

**Authors:** Anne-Françoise de Chambrier, Rachel Sermier Dessemontet, Catherine Martinet, Michel Fayol

**Affiliations:** a University of Teacher Education from State of Vaud, Special Needs Education Unit, Av. de Cour 33, 1014, Lausanne, Switzerland; b University of Clermont Auvergne, LAPSCO CNRS, Av. Carnot 34, 63000, Clermont, France

**Keywords:** Rapid automatized naming, Intellectual disability, Reading difficulties, Pair-matched

## Abstract

**Background:**

A deficit in Rapid Automatized Naming (RAN), acknowledged to be linked to dyslexia, has rarely been investigated as a potential explanation of the reading difficulties that children with intellectual disability (ID) often face. The existing studies mainly focused on adolescent or adults with ID matched to typically developing (TD) children on verbal mental age, or used a single RAN task.

**Aims:**

The aim of this study was to compare the RAN pattern and skills of children with ID and low reading skills to the ones of TD children with matched reading skills.

**Method:**

30 children with mild to moderate ID with mixed etiology (*M* = 9.4 years-old) were pair-matched to 30 TD children (*M* = 4.3 years-old) on phonological awareness- and reading-level. They were all administered color, object, finger, and vowel RAN tasks.

**Outcomes and results:**

Results showed that children with ID had more domain-specific RAN skills and were largely slower in most of the RAN tasks than their younger TD peers.

**Conclusions and implications:**

This suggests that a deficit in RAN should be added to the explanations of their frequent reading difficulties, which might open new remediation possibilities.

**What this paper adds?**

Recently, a growing body of work has sought to understand why children with intellectual disability (ID) often face difficulties in learning to read. It has been highlighted that phonological awareness (PA) – known to be involved in typical reading acquisition and in dyslexia – is also an area of weakness among children with ID. It has even been shown that children with ID exhibit a deviant developmental pattern of PA. Rapid Automatized Naming (RAN), which has also been deeply studied among typically developing (TD) children and among children with specific learning disorders, has conversely been far less investigated among individuals with ID. First, the few studies that compared the RAN skills of individuals with ID to the ones of TD children led to contradictory results regarding the preservation or impairment of these skills. Since RAN skills have been shown to follow an asymptotic evolution, the age of the individuals with ID and of the TD children to whom they are matched might be crucial. Second, it has not yet been investigated whether the RAN developmental pattern of children with ID is similar or different from the one of TD children. Third, most existing research has assessed RAN skills in children who already have reading skills, making direction of influence hard to ascertain. This study examined the RAN skills of 6- to 12-year-old individuals with ID with very low reading skills on four RAN tasks relative to the ones of TD children matched on PA- and reading-level, which has not yet been done. It shows that children with ID are characterized by both an atypical developmental pattern as well as by a deficit in RAN.

## 1 Introduction

In the past decades, the processes underlying reading acquisition have been amply studied among typically developing (TD) children and among children facing learning disabilities. For example, it is largely acknowledged that phonological awareness (PA) and letter-sound knowledge are positively and strongly related to reading acquisition, and that weaknesses in these skills are implicated in the learning disorder faced by dyslexic children (Swan and Goswami, 1997). It has also been highlighted that children with an intellectual disability (ID), who often face reading difficulties, exhibit both a deviant developmental pattern and weaknesses in PA (Channell et al., 2013; Lemons et al., 2013). The contribution of Rapid Automatized Naming (RAN) in reading acquisition and in reading difficulties has also been amply studied among TD children and among children with specific learning disabilities (Araújo et al., 2015; Georgiou et al., 2013a; Landerl et al., 2019; Wolf and Bowers, 1999). In contrast, the RAN skills of individuals with ID were much less studied. They were mainly investigated among adolescents or adults matched to TD children on verbal mental age, and the few results that emerged were inconsistent (Barker et al., 2013; Leite dos Anjos et al., 2019; Saunders and DeFulio, 2007; Soltani and Roslan, 2013). Moreover, when RAN skills were investigated among children with ID with a comparison group of TD children, this was only done through a single RAN task (van Tilborg et al., 2014), preventing the study of the RAN developmental pattern. The current study aimed at investigating the RAN pattern and skills of children with ID on the basis of several RAN tasks administered to children with ID and low reading skills to TD children pair-matched on reading- and PA-level.

## 2 Theory

### 2.1 Development of RAN skills and their links to reading (dis)ability

RAN refers to the ability to name as fast as possible a sequence of highly familiar visual stimuli like objects, letters, or digits (Denckla and Rudel, 1974). This apparently simple activity requires many different cognitive skills such as attention to the stimuli, visual and integration processes, access and retrieval of phonological codes, serial processing, articulation, and processing speed (Georgiou et al., 2013b; Hornung et al., 2017a; Manis et al., 1999; Norton and Wolf, 2012; Wolf and Bowers, 1999). The concept of RAN was first introduced by Geschwind and Fusillo in 1966 (Geschwind and Fusillo, 1966) after which Denckla and Rudel (1972, 1974, 1976) initiated a series of studies that established RAN as a predictor of reading success (Denckla and Cutting, 1999). They began with a color naming task in which participants had to name as fast as possible 50 squares of five primary colors repeated in random order (Denckla and Rudel, 1972) and found that the speed with which color names were retrieved differentiated poor from normal readers. They then developed additional tasks of RAN (digits, letter and objects naming) and found again that the speed with which these items were named was predictive of reading achievement (Denckla and Rudel, 1974). It is now well established that RAN is related to reading success (Araújo et al., 2015; Kirby et al., 2010), in both alphabetic and non-alphabetic writing systems (Landerl et al., 2019; Siddaiah and Padakannaya, 2015). RAN skills are more particularly linked to reading fluency (Cirino et al., 2005; Georgiou et al., 2016) and to a lesser extent to reading accuracy (Neuhaus et al., 2001). RAN association with reading is at least partially mediated by letter knowledge and PA (Torgesen et al., 1997; Wagner et al., 1994). For example, through a large-scale study conducted from kindergarten to first grade, Poulsen et al. (2015) reported that PA and letter knowledge explained from 18 to 56% of the RAN-reading relationship. However, RAN was also found to contribute to reading performance after controlling for PA and letter knowledge (Kirby et al., 2003; McBride-Chang and Manis, 1996; Scarborough, 1998).

The structure of RAN depends on the subtests that are administered and seems to evolve with time. After several month of formal education, a distinction is usually made between alphanumeric RAN—the rapid naming of familiar written symbols, such as letters and digits—and nonalphanumeric RAN (Denckla and Rudel, 1974; Donker et al., 2016; Savage et al., 2008; van den Bos et al., 2003). For example, Donker et al. (2016) showed that among a sample of 133 children with a mean age of 8:11 years, a two-factor model with alphanumeric items (digits, letters) separated from nonalphanumeric items (colors, pictures) fitted the data significantly better than the one general factor model. Relations are consistently found to be stronger between reading and alphanumeric RAN rather than between reading and nonalphanumeric RAN (Bowey, 2005; Norton and Wolf, 2012). With progress in formal education and increasing exposure to print-to-sound correspondences during word and number reading activities, naming written symbols becomes faster than naming nonalphanumeric stimuli (Denckla and Rudel, 1974; Clayton et al., 2020; Norton and Wolf, 2012). Conversely, at the beginning of education, the ability to quickly name different stimuli such as colors, objects, or letters relies mainly on a single common ability. Hornung et al. (2017a) investigated the structure of RAN on the basis of seven types of items (colors, objects, vowels, consonants, digits, dice, and finger configurations) administered to 122 children in the fall of their first grade. The authors concluded that rapid naming in children was best explained by the model investigating a unique and general RAN factor. In kindergarten, the most commonly administered RAN tasks are color and object RAN, which strongly correlate with each other (Georgiou et al., 2013b). In a paper entitled “The power of vowels”, Hornung et al. (2017b) recently reported that vowels RAN administered at kindergarten was a strong and unique predictor for reading accuracy in first grade.

Underperformance in RAN has been proposed to be an additional explanation of developmental dyslexia, beyond the well-established role played by a deficit in PA (Araújo and Faísca, 2019). Indeed, according to the double-deficit hypothesis, PA and RAN are two separable sources of reading dysfunction and the cumulation of deficits cause more significant reading difficulties than a single deficit (Norton et al., 2014; Torppa et al., 2017; Wolf and Bowers, 1999). Moreover, dyslexic children do not experience the growing ease observed among their TD peers in quickly naming frequent visual symbols (letters and digits) compared to less frequent ones (colors and objects) (Norton and Wolf, 2012). Consequently, dyslexic children are better characterized by a deficit in alphanumeric compared to nonalphanumeric RAN. This is all the more so from around 9 years of age, after a few years of formal education that widen the gap between them and normal readers in terms of alphanumeric automatization (Cohen et al., 2018; Kirby et al., 2003; Lervåg and Hulme, 2009).

### 2.2 Intellectual disability and reading

ID is characterized by impaired intellectual functioning (IQ under 70–75) in addition to deficits in adaptive behavior that affect everyday living (Schalock et al., 2010). Several studies point out that individuals with ID often struggle to learn to read and that a significant number of them are illiterate (Lemons et al., 2013; Ratz and Lenhard, 2013; Towles-Reeves et al., 2008). Part of the learning difficulties faced by children with ID is explained by extrinsic factors, such as poor home literacy practices (Light and Kelford Smith, 1993) and limited reading instruction at school (Ahlgrim-Delzell and Rivera, 2015; Kliewer and Biklen, 2001; Ruppar, 2015). Other studies have also highlighted intrinsic difficulties by investigating the cognitive processes involved in reading acquisition among ID children and their developmental pattern. On the one hand, the longitudinal predictors of reading performance are not very different among children with ID than among TD or dyslexic children. PA and letter-sound knowledge were found to be significant predictors of word identification and reading comprehension in different profiles of children with ID (Kennedy and Flynn, 2002; Laing et al., 2001; Laws and Gunn, 2002; Levy et al., 2003; Menghini et al., 2004; Sermier Dessemontet and de Chambrier, 2015; Snowling et al., 2002). On the other hand, several studies indicate that PA is an area of weakness in children with ID of different etiologies (Adlof et al., 2015; Laing et al., 2001; Lemons and Fuchs, 2010; Menghini et al., 2004; Sermier Dessemontet et al., 2017). PA of children with ID is also marked by an atypical pattern of development. Indeed, it has repeatedly been found that these children especially struggle in rhyme awareness while achieving some phonemic tasks better (e.g., first phoneme detection) in contrast to their TD peers (Sermier Dessemontet et al., 2017; Snowling et al., 2002; Steele et al., 2013).

### 2.3 Intellectual disability and RAN

Relative to PA and letter-sound knowledge, fewer studies examined the RAN performance of individuals with ID or its relation to reading acquisition (Barker et al., 2013; Channell et al., 2013; Laing et al., 2001; Leite dos Anjos et al., 2019; Levy et al., 2003; Saunders and DeFulio, 2007; Soltani and Roslan, 2013; Temple et al., 2002; van Tilborg et al., 2014; Ypsilanti et al., 2006). Most of the studies were conducted with samples including mainly adolescents or adults (Channell et al., 2013; Laing et al., 2001; Leite dos Anjos et al., 2019; Levy et al., 2003; Saunders and DeFulio, 2007; Soltani and Roslan, 2013; Temple et al., 2002; Ypsilanti et al., 2006). On the one hand, the available works suggest that among this population, as well as for TD children, RAN constitutes a dimension *per se* (Barker et al., 2013), is related to reading skills (Barker et al., 2013; Laing et al., 2001; Saunders and DeFulio, 2007; Soltani and Roslan, 2013; but see Levy et al., 2003), and contributes in reading performance above PA (Soltani and Roslan, 2013). On the other hand, studies that compared the RAN skills of ID individuals to those of TD children produced inconsistent results. Laing et al. (2001) did not find any significant difference for individuals with Williams syndrome (aged 9–27 years) compared to TD children (aged 6.9 years). Neither did Channell et al. (2013) for adolescents (aged 12.6–19.3 years) with mixed-etiology ID compared to TD children (aged 8.6 years). Ypsilanti et al. (2006) found that relative to TD children (aged 6.1 years), individuals with Williams syndrome (aged 10.2–17.8 years) were slower in naming colors and made more errors in naming pictures, while participants with Down syndrome (aged 14.2 to 14.11 years) were not significantly slower but made more errors in naming words and pictures. Leite dos Anjos et al. (2019) found that youth with ID (aged 6–16 years) were significantly slower in RAN than dyslexic children, suggesting that the differences would have been more important if the RAN skills of the ID group had been compared to the ones of TD children. Such a significant and important difference has been found in the only study that focused on young children with ID (van Tilborg et al., 2014). These researchers found that the ID group (7.6 years) was more than two times slower than the TD group (6 years).

### 2.4 The current study

The current study aimed at investigating the RAN pattern and skills of children with ID with very low reading skills in comparison to TD children matched on reading and PA levels, which has not yet been done so far. Indeed, the RAN skills first have to be further studied among children with ID. The observation that RAN skills appear to be relatively preserved among adolescents or adults with ID could be accounted by the procedures that are generally used to match individuals with ID to TD ones, coupled with the asymptotic evolution that has been found for RAN in typical development. Indeed, when comparing individuals with ID and TD on any skills, individuals with ID are always matched to younger TD individuals. Yet, van den Bos et al. (2002) showed that among TD individuals, important gains in speed of naming were made up to age 10, while fewer gains occurred after age 12 and while a maximum speed was reached for most of the RAN tasks around age 16. Thus, it could be that no difference appears when the RAN skills of adolescent/adults with ID – who are probably near or at their maximum speed of naming – are compared to the RAN skills of TD children, whose speed of naming is in full evolution. In contrast, differences could be more important when comparing the skills of children with ID (below age 12), whose speed of naming is in full evolution, to the ones of matched (younger) TD children. However, the only study that compared the RAN skills of children with ID to the ones of TD children (van Tilborg et al., 2014) comprised children with IQs up to 85 in the ID sample and included a single measure of (objects) RAN. Therefore, research is clearly lacking regarding the RAN skills of children with ID.

Second, the skills of children with ID have to be investigated on several tasks of RAN. In addition to the reliability problem resulting from the single RAN task used in Van Tilborg et al.’s study (2014), this did indeed not allow to study the pattern of RAN skills in children with ID relative to TD children. Since a deviant developmental trajectory has been found for PA in children with ID (Sermier Dessemontet et al., 2017), and since data highlighted differences only in some specific RAN tasks in adolescents with ID (Ypsilanti et al., 2006), the RAN skills of young children with ID should be investigated on several types of items in order to test whether their RAN pattern is similar to that of TD children.

Finally, the RAN skills of children with ID and with very low reading skills should be examined and compared to the skills of TD children matched on reading and PA levels. Indeed, in previous studies, the ID group has often been matched to TD children according to their verbal mental age (Channell et al., 2013; Ypsilanti et al., 2006), and most existing research has assessed RAN skills in children who already have reading skills. This makes direction of influence hard to ascertain since skills such as PA or RAN are not only reading precursors but also develop under the influence of reading acquisition (Perfetti et al., 1987; Powell and Atkinson, 2020). To prevent these issues, it is important to select and to match ID and TD children on the basis of low reading skills. Indeed, any differences that could be found in RAN between reading-disabled individuals with ID and TD children with higher reading skills could be interpreted as a result rather than as a cause of such differences (Bryant and Goswami, 1986). Conversely, if the two groups of children are at the same and low reading level, any differences in RAN between them can be considered as causally related to the reading disabilities (Bryant and Goswami, 1986; Manis et al., 1996). Furthermore, since RAN association with reading is partially mediated by PA, ID children should also be matched to TD children according to their PA level. Indeed, if TD children had similar reading skills but higher PA abilities than ID children, their possible higher RAN skills could be attributed to stronger phonological representations rather than to faster access to them. In contrast, comparing the RAN skills of children with ID to reading- and PA-level matched TD children allows to impute the possible differences to an original deviance in RAN rather than to low exposure to reading or to weak phonological representations. If such a RAN deficit is confirmed, it should be added to the reasons why children with ID often face difficulties in reading.

## 3 Material and method

### 3.1 Participants

For the current study, a subsample of another study conducted in the French-speaking region of Switzerland on reading instruction for students with ID with mixed etiologies (Sermier Dessemontet et al., 2021) was pair-matched to children attending kindergarten classes in France. The original study included 48 children with ID who knew at least three letter-sound correspondences without being able to decode syllables (Sermier Dessemontet et al., 2021). For the current study, children with autism spectrum disorder were excluded, since this disorder has been found to affect RAN skills (Losh et al., 2010). This left the ID sample to include 33 students. TD children were then recruited in French kindergarten classes on the basis of their scores at the reading and PA tasks (Moser and Berweger, 2007, see details below). According to the levels determined by this test, almost all children with ID had the lowest level in reading (knowing some capital letters) and in PA (being at best able to blend two or three syllables into simple words). Two children with ID had the lowest level in reading while having a level 2 in PA (the ability to recognize and distinguish oral units such as rimes or syllables, to segment simple and familiar words into two syllables, and to blend the first phoneme to the rest of a word). One child with ID had a level 2 in reading (the ability to recognize all the vowels and the large majority of the consonants, mainly in their capital forms) as well as in PA.

In order to be included in the study, TD children could not have any visual or hearing impairments, neurological or developmental disorders, or speech impediments requiring therapy support. Recruitment was done in the first and second grade of kindergarten, until the tested TD kindergartners matched each child with ID on reading and PA levels.

After having removed three extreme outliers in RAN skills (two ID and one TD children), the final sample was composed of 30 children with ID matched to 30 TD children. The ID sample was composed of 13 boys and 17 girls, from 6 to 12 years old (*M* = 9.4, *SD* = 1.9). Their nonverbal IQ ranged from 40 to 72, and the majority of the participants (63%) had a moderate ID (IQ < 50). The participants also displayed significant limitations in adaptive behavior (*M* = 57.6, *SD* = 12). Approximatively a third of the participants had specific syndromes: Down syndrome (n = 5), Williams syndrome (n = 2), Potocki-Lupski syndrome (n = 1), or Tetrasomy 18p (n = 1). The TD sample was composed of 14 boys and 16 girls aged from 3.4 to 5.3 (*M* = 4.3, *SD* = 0.5). Written parental consent was received for all participating children. There is no ethical committee in the institution in which this study was conducted but the ethical code of the Universities of Teacher Education of the country was followed.

### 3.2 Measures

In order to match children with ID to TD children on the basis of their reading and PA levels, the French translation (Moser et al., 2004) of a standardized criterion-referenced comprehensive battery of academic achievement tests (Moser and Berweger, 2007) was used. This battery was designed to measure the progress of Swiss children in literacy and mathematics from the beginning of kindergarten (4 years old) to the end of first grade (7 years old). The literacy part of the test comprises subtests of phonological awareness, letter-sound knowledge, nonword and word reading, and written comprehension. It was constructed on the basis of the Item Response Theory with a sample of over 1,000 TD students. In the current study, only the subtests of phonological awareness, letter-sound knowledge, and non-word and word reading were used. The original tests are in German (Moser and Berweger, 2007), but were translated to French in an unpublished version used for research (Moser et al., 2004). This French version was administered to children with mild and moderate ID in previous studies and showed that the internal reliability of the subtests was high (Cronbach's α = 0.91–0.98) (Sermier Dessemontet and de Chambrier, 2015; Sermier Dessemontet et al., 2021). Item difficulty and discrimination index were also found to be appropriate (Sermier Dessemontet and de Chambrier, 2015).

#### 3.2.1 Reading level

*Letter sound naming test*. The letter sound naming test is comprised of 31 items. Children are first shown capital letters, then lowercase letters. They are required to say each letter's sound. The reliability of this test is high for children with mild and moderate ID (Cronbach's = 0.97) (Sermier Dessemontet and de Chambrier, 2015). *Nonword and word reading test*. This test is comprised of two subtests: 12 nonwords with a consonant-vowel (CV) structure and 4 nonwords with a CVC, CVCV, or CCVC structure; and 14, high-frequency regular words with various structures (e.g., CVCV, CVCC, CVCCV, CVCCVC, CCVCVCVC) such as moto (motorcycle), livre (book), tableau (painting), journal (journal), and crocodile (crocodile). Each subtest was stopped after seven consecutive failed items. The reliability of the test is high for children with mild and moderate ID (Cronbach's = 0.98) (Sermier Dessemontet and de Chambrier, 2015). On the basis of the Item Response Theory, the test defines five levels of reading abilities, with the two first levels relevant for the current study. At the lowest level (up to 17 points), children are, at best, able to recognize some capital letters. At level 2 (up to 27 points), all vowels are recognized as well as a large majority of consonants, mainly in their capital forms, but children are not yet able to decode, an ability that is characteristic of the following level.

#### 3.2.2 Phonological awareness level

The phonological awareness test comprises nine phonological awareness subtests: segmentation of words in syllables (7 items), blending syllables to form words (6 items), rhyme detection (6 items), first phoneme blending (5 items), blending phonemes to form words (8 items), first phoneme detection (8 items), last phoneme detection (8 items), segmenting words in phonemes (6 items), and phoneme substitution (6 items). For standardization purposes, items were recorded and presented to the children through computer loudspeakers. Each subtest was stopped after five consecutive failed items. A composite score of phonological awareness was calculated with a summation of all the correct answers. The reliability of this test is high for children with mild and moderate ID (Cronbach's = 0.95) (Sermier Dessemontet and de Chambrier, 2015). The PA levels defined by the test follow: At the lowest level (up to 17 points), children are, at best, able to blend two or three syllables to form simple and familiar words. At level 2 (up to 27 points), children are able of analysis activities in addition to the blending ones; they can recognize and distinguish oral units such as rimes or syllables, they can segment simple and familiar words into two syllables, and they can blend the first phoneme to the rest of a word.

#### 3.2.3 Rapid automatized naming

The RAN skills of the children were assessed through four naming subtests developed from the work of Denckla and Rudel (1974) as well as of Hornung et al. (2017a). Children were required to name as fast as possible five recurring *colors* (black, blue, green, red and yellow), *objects* (dog, foot, tree, book and table), *capital vowels* (E, A, O, I, and U), and *finger-numeral configurations* (1–5 fingers). For all tasks, items were serially and randomly displayed in two 5 × 5 grid on horizontally presented A4 sheets. The tasks were only administered if the children were able to name the five items in the training phase that preceded each subtest. In order to avoid a lengthy duration of the tests' administration—especially for the ID group—the score was the number of items correctly named within a 30-second limit as defined by an electronic chronometer. The reliability of the measure is high (Cronbach's = 0.90) (Hornung et al., 2017a).

#### 3.2.4 Nonverbal intelligence test

The cognitive abilities of the children with ID were assessed with the Culture Fair Intelligence Test, Scale 1 revised (CFT 1-R) (Weiss and Osterland, 2013). The CFT 1-R has high reliability coefficients for internal consistency and test-retest reliability (*r* = 0.95–0.97). The manual also provides evidence for inner validity and convergent validity.

#### 3.2.5 Adaptive behavior assessment

The adaptive behavior of the ID participants was measured with the French version of the ABAS-II (second edition, 5–21 years; Harrison and Oakland, 2003). The teacher form was used and was completed by the main teacher of each child with ID. The teacher form starts with the instructions for completing the questionnaire. The ABAS-II has high reliability coefficients for internal consistency, test-retest, and inter-rater reliability (0.8–0.9). The manual provides solid evidence of convergent and divergent validity. The French version of the ABAS-II was found to have high internal reliability in a previous study conducted with students with mild and moderate ID (Cronbach's = 0.8–0.9) (Sermier Dessemontet, Bless and Morin, 2012).

### 3.3 Procedure

The tests were individually administered to each child in the fall in a quiet room of their respective schools. The nonverbal intelligence test and the Adaptive Behavior Assessment System – second edition (ABAS-II) questionnaire were administered only to children with ID, in order to assess the severity of their intellectual disability. All the tests (letter sound naming, nonword and word reading, phonological awareness, Rapid Automatized Naming, nonverbal intelligence) were administered by four graduate students with a bachelor's degree in psychology or in speech therapy and the ABAS-II questionnaire was completed by the teachers during the fall. The explanations and instructions included in the tests administered to the children were given in an *easy to understand language* in order to ensure that both the children with ID and the young TD children understood what they had to do. The instructions for the administration of each test (tasks explanations, verbal instructions, sample items, stopping criteria) were very clearly specified in the test protocols and the graduate students were trained to administer each test during a half a day workshop by our research team. Both groups of children began with the reading and then the PA tasks, followed by the RAN tasks. The testing session lasted approximately 30 min for the TD children and the children with ID. A second session of approximately 30 min was required in order to administer the nonverbal intelligence test to the children with ID.

## 4 Results

### 4.1 Descriptive statistics

Table 1 displays the descriptive statistics regarding age and RAN tasks for both groups of children and the IQ for children with ID. The TD group achieved color and object naming very well, whereas vowel naming showed the lowest mean score. Children with ID also achieved color and object naming the best but for them finger naming was less successful and showed the highest standard deviation.

**Table 1:** Descriptive statistics for age and rapid automatized naming (RAN) tasks for both groups of children (TD = typically developing children; ID = children with intellectually disability) and for IQ for the ID group.

	TD (n = 30)				ID (n = 30)			
	*Mean*	*SD*	*MIN*	*MAX*	*Mean*	*SD*	*MIN*	*MAX*
Age	4.31	0.51	3.4	5.3	9.43	1.89	6.2	12.5
RAN colors	20.03	5.37	10	29	14.63	4.87	4	25
RAN objects	19.27	4.83	10	27	12.83	7.67	0	25
RAN vowels	12.57	9.56	0	31	10.07	7.71	0	27
RAN fingers	16.93	7.23	0	27	8.27	9.78	0	35
IQ					48.43	9.25	39	72

### 4.2 Correlations

Correlations between each RAN subtest and age within both groups of children – and with IQ within the ID group – are displayed in Tables 2 and 3. The correlational patterns were different across the groups. Notably, in the ID group, no RAN task correlated with age nor with IQ score.

**Table 2:** Correlations between each rapid automatized naming (RAN) subtest (color, object, vowel, and finger naming) and age among typically developing (TD) children.

	RAN colors	RAN objects	RAN vowels	RAN fingers	Age
RAN colors					
RAN objects	.663∗∗				
RAN vowels	.250	.291			
RAN fingers	.362∗	.526∗∗	.408∗		
Age	.048	.146	.447∗	.436∗	

∗∗ = significant at .01 level; ∗ = significant at .05 level.

**Table 3:** Correlations between each rapid automatized naming (RAN) subtest (color, object, vowel, and finger naming), age and IQ among children with intellectual disability (ID).

	RAN colors	RAN objects	RAN vowels	RAN fingers	Age	IQ
RAN colors						
RAN objects	.192					
RAN vowels	.142	.123				
RAN fingers	.328	.088	.225			
Age	-.013	.163	.209	.042		
IQ	.258	-.088	-.104	.175	-.612∗∗	

∗∗ = significant at .01 level; ∗ = significant at .05 level.

Among TD children, most of the correlations between the four RAN subtests were significant and ranged from moderate to strong (except between vowel and color naming and between vowel and object naming). Conversely, there was no significant correlation between the four RAN subtests among children with ID.

### 4.3 Differences in RAN skills between the two groups of children

Given the absence of links between the four RAN subtests in the ID group, the differences in RAN skills across the groups were analyzed on each subtest score rather than on a composite score. In order to compare the RAN skills of children with ID to the ones of TD children, a Student test (t) was used when the distribution of both groups was normal according to a Shapiro-Wilk test and when variance was homogeneous across the groups according to a Levene's test. As the distribution of one of the groups was not normal and/or when homogeneity of variance was violated, the differences across the groups were tested through a non-parametric Mann-Whitney test (U). Table 4 displays the test used, the results and the effect sizes of the differences across the groups for each RAN subtest. The differences between the TD and the ID group were significant and large for color, object, and finger naming, whereas the difference did not reach significance for vowel naming.

**Table 4:** Differences in rapid automatized naming (RAN) tasks across the typically developing (TD) group and the group with intellectual disability (ID).

	TD (N = 30)		ID (N = 30)		*t* or U	*sig.*	*d*
	*Mean*	*SD*	*Mean*	*SD*			
RAN colors	20.03	5.37	14.63	4.87	*t(58)* = 4.08	.000	1.05
RAN objects	19.27	4.83	12.83	7.67	*t(58)* = 3.89	.000	1
RAN vowels	12.57	9.56	10.07	7.71	U = 369.50	.229	0.29
RAN fingers	16.93	7.23	8.27	9.78	U = 208	.000	1

## 5 Discussion

This study investigated the RAN pattern and skills among children with ID who struggle to read compared to reading- and PA-level matched TD children. It aimed at investigating whether a deficit in RAN processing could be involved in the reading difficulties faced by children with ID. Our results first indicated that among children with ID (6.2–12.5 years old), no RAN task correlated with age, despite the relatively large age span. This indicates that among this group, the degree of familiarity with the items was not related to the number of school years attended by the children. RAN skills did not either correlate with IQ score, suggesting that RAN performance does not depend on the severity of the ID.

Within the different RAN skills, children with ID achieved color and object naming better than other tasks, as did TD children. However, in contrast to their peers, they achieved finger naming worse than vowel naming. This result could be due to the fact that rapid naming of finger configurations is related to subitizing (Hornung et al., 2017a), an ability that has been found to be impaired in children with ID (Jimenez and Saunders, 2019; O’ Hearn et al., 2011).

Regarding the interrelations between the four RAN tasks, no significant correlation was found among children with ID, whereas most of the RAN tasks correlated with each other among TD children. This suggests that RAN skills are more domain-specific among children with ID than among TD children. One could argue that the correlations between RAN tasks were weaker among children with ID because of their older age, given that the structure of RAN has been found to evolve from a single common factor to the alphanumeric and the nonalphanumeric factors (Hornung et al., 2017a; van den Bos et al., 2002). However, while previous factorial analyses effectively highlighted separated RAN factors after age 8, the rapid naming of different types of items was still found to correlate up to a late age among TD individuals. For example, van den Bos et al. (2002) reported correlations ranging from .52 to .65 between color and picture naming among 8-, 10-, 12-, 16-, and even 46-year-old individuals. Thus, the absence of correlation found among our participants with ID suggests that their RAN skills are more item-specific than among TD individuals from childhood. Altogether, the findings suggest that there are more differences than similarities between the pattern of RAN skills of children with ID and TD children.

The differences in RAN skills between the two groups of children were investigated on each RAN subtest rather than on a composite score, given the absence of correlation between the RAN tasks among children with ID. Interestingly, the group differences were highly significant for color, object and finger naming, but not for vowel naming. Consequently, our findings confirm and extend those found in the only previous study that focused on children with ID. While van Tilborg et al. (2014) highlighted that ID children of 7.6 years were much slower than TD children of 6 years on their single RAN task (object naming), our study highlights very significant differences between the ID group of 9.4 years and the TD group of 4.3 years on three of the four RAN tasks. The current findings are therefore clearly in favor of a RAN deficit among children with ID who attend primary school. Longitudinal studies could investigate whether individuals with ID also exhibit the strong progress in RAN that has been observed in TD participants up to adolescence, which could explain why the differences in RAN are less noticeable when adolescents with ID are compared to TD children (Channell et al., 2013; Laing et al., 2001; Ypsilanti et al., 2006).

It should be noted that the difference between TD children and those with ID was not significant for vowel naming, but this was due more to the low skills of the young TD children than to preserved skills among children with ID. Capital vowels were chosen because they are easier to produce and more familiar to young children (Hornung et al., 2017b). Such items were probably also easier to name for the children with ID than consonants or even lower-case vowels. If a sample of less struggling ID readers could be matched on reading-level to older TD children in order to administer a consonant RAN to both groups, the difference between the groups may also be significant for letters. In any case, it can be highlighted that the ID group of the current study (mean age of 9.4) did not show a higher automatization degree for alphanumeric items relatively to nonalphanumeric ones, which is generally reported around age 9 among TD children (Norton and Wolf, 2012).

Some limitations should be considered in the current study. First, as explained in the Procedure section, the students who administered the tests to the children were trained by our research team. However, the reliability with which they administered the test was not measured. Further studies could do it in order to ensure that the experimenters administer the tests the same way and in the manner prescribed in the protocols. Second, the procedure followed to match children with ID to TD children took the reading as well as the PA-level into consideration, but not gender. Further studies could include this variable into the matching procedure to ensure that the observed differences in RAN are not linked to gender.

## 6 Conclusion

In sum, this study – that focused on children with mild to moderate ID and with low reading skills – shows that RAN skills follow a different pattern among these children compared to TD children. It also reveals that a RAN deficit should be added to other explanations already identified to account for the frequent reading difficulties faced by children with ID. Since a few studies showed that RAN skills could successfully be trained (Conrad and Levy, 2011; Fugate, 1997; Vander Stappen and van Reybroeck, 2018), such investigations among children with ID could open new perspectives to improve their reading acquisition.

## Declarations

### Author contribution statement

Anne-Francoise de Chambrier: Conceived and designed the experiments; Performed the experiments; Analyzed and interpreted the data; Contributed reagents, materials, analysis tools or data; Wrote the paper.

Rachel Sermier Dessemontet, Catherine Martinet: Performed the experiments; Contributed reagents, materials, analysis tools or data.

Michel Fayol: Conceived and designed the experiments; Analyzed and interpreted the data; Contributed reagents, materials, analysis tools or data.

### Funding statement

This work was supported by The Swiss National Science Foundation (100019_173096).

### Data availability statement

Data will be made available on request.

### Declaration of interests statement

The authors declare no conflict of interest.

### Additional information

No additional information is available for this paper.
